# Receipt of guideline-concordant care and survival among young adult women with non-metastatic breast cancer

**DOI:** 10.1007/s10549-024-07570-w

**Published:** 2024-12-09

**Authors:** Manami Bhattacharya, Benmei Liu, Allison W. Kurian, Jennifer Stevens, Lindsey Enewold, Dolly C. Penn

**Affiliations:** 1 Surveillance Research Program, Division of Cancer Control and Population Sciences, National Cancer Institute, Rockville, MD USA; 2 Healthcare Delivery Research Program, Division of Cancer Control and Population Sciences, National Cancer Institute, Rockville, MD USA; 3 Stanford University School of Medicine, Palo Alto, CA USA; 4 Information Management Services, Inc, Rockville, MD USA; 5 Bethesda Community Clinic, Canton, GA USA; 6 Division of Clinical and Health Services Research, National Institute On Minority Health and Health Disparities, National Institutes of Health, 6707 Democracy Boulevard, Suite 800, Bethesda, MD 20892-5465 USA

**Keywords:** Breast cancer, AYA, Guideline-concordant care, Survival, TNBC

## Abstract

**Purpose:**

Adolescent and young adults (AYA) with breast cancer, compared to older adults, are diagnosed with more aggressive cancers, at more advanced stages and may undergo more aggressive treatment but have worse survival. Despite this, no research has studied the effects of the receipt of National Comprehensive Cancer Network (NCCN) defined guideline-concordant care (GCC) for breast cancer on AYA survival. We examined the association of GCC with survival among young adult (20–39 years old) breast cancer survivors.

**Methods:**

We used the Patterns of Care Study; a stratified random sample of 952 young adult women diagnosed with Stage I–III breast cancer in 2013. NCCN guidelines were used to categorize treatment as GCC or non-GCC. We used Kaplan–Meier curves, log-rank tests, and Cox-proportional hazards models to evaluate the effect of GCC on breast cancer-specific survival, stratifying by triple-negative breast cancer (TNBC) and non-TNBC, and adjusting for sociodemographic and clinical factors.

**Results:**

All univariate analyses showed that non-GCC was associated with worse survival than GCC. The association was statistically significant for non-TNBC (Hazard ratio: 3.45, CI 1.64–7.29) and TNBC (Hazard ratio: 3.70, CI 1.02–13.43) in multivariable Cox models adjusted for sociodemographic variables and for non-TNBC (Hazard ratio: 3.13, CI 1.13–8.72) when the model was adjusted for sociodemographic and clinical variables.

**Conclusion:**

Among young adult women with non-metastatic breast cancer, while receipt of NCCN GCC is univariately associated with better survival for both TNBC and non-TNBC, the effect of sociodemographic and clinical factors on the association differs by TNBC status. Further investigation with larger TNBC samples is needed.

## Introduction

Breast cancer accounts for 30% of cancers among adolescent and young adult (AYA) women (15–39 years old) in the United States and is the most common type of cancer diagnosed in this age group [1]. AYA breast cancer is more likely to be diagnosed in advanced stages, with more aggressive subtypes, and is more likely to be associated with inherited genetic mutations [1]. Survival after breast cancer in AYA women tends to be shorter than in other age groups, even accounting for the higher prevalence of worse-prognosis cancer subtypes [2–4]. Prior studies estimate that AYA breast cancer survivors with early-stage disease are 39% more likely to die of their cancer compared to women who are diagnosed later in life [5].

Strategies to improve survival among women of all ages with breast cancer include early detection and guideline-concordant cancer care. The National Comprehensive Cancer Network (NCCN) treatment guidelines specify best practices for breast cancer treatment. NCCN guideline-concordant care (GCC) improves survival in general populations of women with breast cancer, but the treatment guidelines are not age specific; instead, the NCCN focuses on cancer biology in the treatment recommendations and provides separate supportive care guidelines for AYA survivors [6].

Few studies focus on evaluating treatment and survival among women under 40 years old. Murphy et al. (2019) [7] found that AYA patients had higher use of more aggressive cancer therapies; Fredholm et al. (2009) [8] found younger age to be associated with worse survival, even after accounting for tumor biology and treatment; and DeRouen et al. (2013) [9] found that Black and Hispanic AYA survivors were less likely to receive guideline-concordant radiation and experienced worse survival. In a study of AYA survivors, Keegan et al. (2013) [10] found that short-term survival varies: Black women, women with triple-negative breast cancer (TNBC) or hormone receptor (HR)-negative and human epidermal growth factor receptor 2 (HER2)-positive (HR − /HER2 +) cancers, women of lower socioeconomic status, and publicly insured women experienced shorter survival. However, there has been little to no focus on whether receipt of GCC is associated with longer survival among breast cancer survivors under 40 years old, after considering other relevant sociodemographic and clinical factors.

Understanding the effect of GCC on cancer-specific survival among women under 40 years old is important given that women diagnosed with breast cancers at this age encounter worse survival and sometimes undergo therapies and treatment plans that are more aggressive than recommended [11]. White et al. (2021) [12] in an analysis examining usage of GCC in young adult women using the Patterns of Care 2013 AYA breast cancer data found that the receipt of GCC is high among young adult women. In the current follow-up study, we examined the association of GCC with survival among young adult breast cancer survivors (20–39 years old), an under-studied population with little known about how GCC affects cancer-specific survival, especially accounting for stage at diagnosis and cancer subtypes.

## Methods

### Data source

We used data from the 2013 Patterns of Care Study (https://healthcaredelivery.cancer.gov/poc/), sponsored by the National Cancer Institute. The Patterns of Care Study (POC) developed a dataset for AYA breast cancer to study treatment patterns in the community. This dataset included a stratified random sample of AYA females diagnosed with breast cancer between January and December 2013, identified from fourteen Surveillance, Epidemiology, and End Results (SEER) cancer registries (Connecticut, Georgia Center for Cancer Statistics, Greater California Regional, Greater Bay Area Cancer Registry (San Francisco-Oakland/San Jose-Monterey), Los Angeles, Seattle-Puget Sound, New Mexico, Iowa, Utah, Detroit Regional, Louisiana, New Jersey, Kentucky, and Hawaii) (https://seer.cancer.gov/data/index.html). AYAs who had a previous cancer diagnosis (other than nonmelanoma skin cancer), a simultaneous diagnosis of a second non-breast primary cancer, or a diagnosis at autopsy/death were ineligible. POC data include tumor biology and detailed treatment information, while SEER includes survival information through 2018, patient demographics, clinical cancer features (such as stage of diagnosis), and a summary of first-course treatment.

### Analytic sample

We included women who were 20–39 years old; no adolescents between 15 and 19 years were identified in the stratified random sample from the diagnosis year 2013 POC study. The included women were diagnosed in 2013 with Stage I–III invasive ductal, lobular, mixed, or metaplastic cancers. We excluded cancers for which HR status, HER2 status, tumor stage, tumor size, or lymph node involvement were either unknown or for which NCCN guidelines were not defined. The final sample included 952 women.

### Measures

#### Outcome

The outcome of this study was breast cancer-specific survival, measured in months, beginning at the month of diagnosis and ending in December 2018, providing at least 5 years of follow-up for all women.

#### Guideline-concordant care

GCC was defined by subtype as a dichotomous (yes/no) variable using NCCN 2013 guidelines because the women were diagnosed in 2013 [13]. GCC was constructed to include receipt of surgery (mastectomy or breast-conserving surgery) and receipt of only the NCCN-recommended systemic therapy for the relevant tumor type. Neoadjuvant and adjuvant systemic therapy were both considered; order of systemic therapy was not considered due to NCCN guidelines allowing for adjuvant chemotherapy regimens to also be considered in a neoadjuvant setting. Clinical trial participation was considered guideline concordant. Lack of surgery or lack of recommended systemic therapy was considered not guideline concordant. A breast cancer oncologist (coauthor A.W.K.) adjudicated patients with unclear guideline concordance [12].

#### Covariates

Covariates included sociodemographic information including age at cancer diagnosis, race/ethnicity, marital status, insurance status (public, private, uninsured), and the census-tract-based Yost quintile as a measure of socioeconomic status (SES) [14]. We also considered American Joint Committee on Cancer (AJCC) stage, and time from diagnosis to treatment initiation (> 8 weeks; ≤ 8 weeks). Charlson comorbidity score was not included as over 85% of women in the cohort had a comorbidity score of 0.

### Statistical Analysis

All analyses were stratified by TNBC and non-TNBC subtypes, as TNBC has worse survival than other cancer subtypes. The Kaplan–Meier method was used to estimate unadjusted cancer-specific survival curves by receipt of GCC (i.e., received GCC versus did not receive GCC), and log-rank test was used to compare the two groups (GCC vs. non-GCC). Cox-proportional hazards models were used to calculate unadjusted and adjusted hazard ratios for non-GCC vs GCC groups. Two multivariable cox models were run for each subtype: Model 1 adjusting for sociodemographic variables and Model 2 adjusting for both sociodemographic and clinical variables. Women lost to follow-up were censored. To address potential immortal time bias, landmarked analyses were considered [15]. We examined the number of deaths occurring within the first 6 months of follow-up across groups and found no breast-cancer deaths occurred. For variance estimation, single primary sampling unit (PSU) within a stratum was treated as certainty PSUs; thus, a single-PSU stratum makes no contribution to the variance. Kaplan–Meier curves, log–rank tests, and Cox models were conducted using the survey survival package in R [16]. All other analyses were conducted in SAS, 9.4 with a critical alpha of 0.05. Institutional review board approval was granted at each SEER registry for POC.

## Results

### Patient characteristics

At the end of the study period, 69.7% of women with TNBC and 80.5% of women with non-TNBC were alive; 11.3% of women with TNBC and 10.4% of women with non-TNBC were lost to follow-up. For both subtypes, almost 42% of women received GCC (Table 1). In both groups, most women were 35–39 years old, non-Hispanic White, partnered, privately insured, and had higher SES. Clinically, most women in both groups were diagnosed as Stage II and received treatment within eight weeks of diagnosis.

**Table 1:** Characteristics of young adult women diagnosed with breast cancer in 2013 by triple-negative breast cancer (TNBC) status, Patterns of Care Study (*n* = 952)

	TNBC (*n* = 195)	non-TNBC (*n* = 757)
	*n* (weighted %)	*n* (weighted %)
Guideline-concordant care (GCC)		
Yes	76 (41.1%)	347 (41.9%)
No	119 (58.9%)	410 (58.1%)
Sociodemographic variables		
Age (Years)		
20–29	40 (22.5%)	124 (17.7%)
30–34	66 (30.6%)	219 (29%)
35–39	89 (46.9%)	414 (53.3%)
Race and/or ethnicity		
Hispanic	37 (25.7%)	131 (20.8%)
Non-Hispanic black	26 (7.8%)	118 (8.9%)
Non-Hispanic white	102 (55.1%)	383 (56.5%)
Other^a^	30 (11.4%)	125 (13.8%)
Marital status		
Not partnered	71 (34.9%)	310 (38.0%)
Partnered	116 (60.1%)	416 (57.6%)
Unknown	8 (5.0%)	31 (4.4%)
Insurance status		
Any private	145 (71.9%)	573 (77.4%)
Public insurance	44 (25.2%)	152 (20.2%)
Uninsured	6 (2.9%)	32 (2.4%)
Yost registry-based quintile		
Lowest	33 (13.2%)	135 (13.4%)
Low medium	38 (21.6%)	128 (17.6%)
Medium	37 (17.9%)	151 (21.5%)
Medium high	43 (22.4%)	154 (22.6%)
Highest	44 (24.9%)	189 (24.9%)
Clinical variables		
Stage at diagnosisb		
I	31 (16.4%)	250 (39.6%)
II	136 (70.3%)	403 (51.6%)
III	28 (13.3%)	104 (8.9%)
Subtype		
HR − /HER2- (TNBC)	195 (100%)	N/A (%)
HR + /HER2 +	N/A (%)	223 (27.5%)
HR + /HER2-	N/A (%)	458 (64.0%)
HR-/HER2 +	N/A (%)	76 (8.5%)
Time from diagnosis to treatment		
≤ 8 weeks	177 (90.8%)	634 (82.1%)
> 8 weeks	18 (9.2%)	123 (17.9%)

*TNBC* Triple-negative breast cancer

*HR* Hormone receptor, *HER* Human epidermal growth factor receptor 2

^a^Other race/ethnic group included Asian or Pacific Islander and American Indian/Alaskan Native, ^b^Derived from the American Joint Committee on Cancer (AJCC) staging system (seventh edition)

### Univariate analyses

**Fig. 1:**
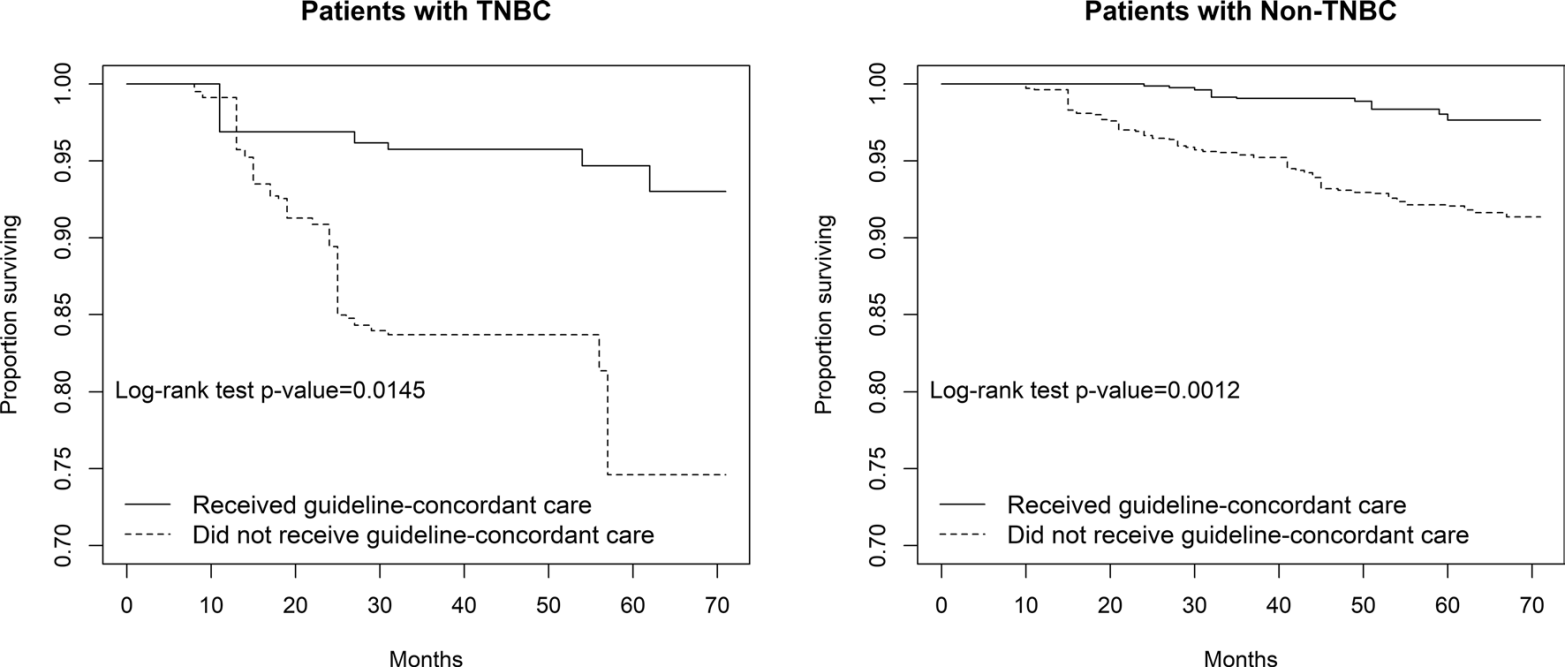
Breast cancer specific survival comparison and log-rank test between guideline-concordant care groups for young adult women (aged 20 to 30y) diagnosed with breast cancer in 2013, stratified by triple-negative breast cancer (TNBC) and non-TNBC subtype, Patterns of Care Study (n = 952)

Kaplan–Meier survival curves and log-rank tests showed among women with non-TNBC, approximately 97% of those who received GCC were estimated to be alive at the end of the study period, compared to 91% of women who did not receive GCC (Fig. 1). Among women with TNBC, about 92% of those who received GCC were estimated to be alive at the end of the study period, compared to 74% of women who did not receive GCC. For either subtype, women who did not receive GCC had a significantly worse survival probability than those who received GCC (log-rank test* p* = 0.0145 for TNBC and *p* = 0.0012 for non-TNBC). Among women who received GCC, those with TNBC had worse survival than women with non-TNBC.

The unadjusted univariate Cox models showed women who did not receive GCC had significantly higher hazards of dying than those who received GCC (HR = 4.08, CI 1.28–12.95 for TNBC; HR = 3.78, CI 1.72–8.28 for non-TNBC) (Table 2).

**Table 2:** Hazard ratios (HR) from univariate Cox models among young adult women diagnosed with breast cancer in 2013 by triple-negative breast cancer (TNBC) status, Patterns of Care Study (*n* = 952)

	TNBC (*n* = 195)	non-TNBC (*n* = 757)
	HR (95% CI)	HR (95% CI)
Guideline-concordant care (GCC)		
Yes	Ref	Ref
No	4.08 (1.28 to 12.95)*	3.78 (1.72 to 8.28)^**^

*TNBC* triple-negative breast cancer, *CI* confidence Interval

–-Unadjusted results

*p-value < 0.05; **p-value < 0.01

### Multivariable Cox models

Multivariable Cox Model 1 adjusted for sociodemographic variables showed women who did not receive GCC still had significantly higher hazards of dying than those who received GCC (HR = 3.70, CI 1.02–13.43 for TNBC; HR = 3.45, CI 1.64–7.29 for non-TNBC) (Table 3). After further adjusting for clinical variables (AJCC stage at diagnosis and time from diagnosis to treatment) in Model 2, women with non-TNBC who received non-GCC still had significantly higher hazard of dying compared to women who received GCC (HR = 3.13, CI 1.13–8.72). The hazard ratio was no longer significant for women with TNBC.

**Table 3:** Hazard ratios (HR) from multivariable Cox models among young adult women diagnosed with breast cancer in 2013 by triple-negative breast cancer (TNBC) status, Patterns of Care Study (*n* = 952)

	TNBC (*n* = 195)		Non-TNBC (*n* = 757)	
	Model 1^a^	Model 2	Model 1	Model 2
	HR (95% CI)	HR (95% CI)	HR (95% CI)	HR (95% CI)
Guideline-concordant care (GCC)				
Yes	Ref	Ref	Ref	Ref
No	3.7 (1.02–13.43)*	1.97 (0.36–10.7)	3.45 (1.64–7.29)**	3.13 (1.13–8.72)*
Demographics				
Age (Years)				
20–29	Ref	Ref	Ref	Ref
30–34	0.74 (0.15–3.54)	1.73 (0.31–9.6)	0.93 (0.42–2.04)	0.96 (0.48–1.92)
35–39	1.39 (0.45–4.30)	2.04 (0.55–7.59)	0.77 (0.38–1.54)	0.67 (0.29–1.53)
Race and ethnicity				
Hispanic	0.64 (0.33–1.26)	0.59 (0.27–1.29)	1.48 (0.6–3.62)	1.07 (0.43–2.65)
Non-Hispanic Black	0.92 (0.17–4.98)	0.84 (0.13–5.4)	3.47 (1.77–6.80)***	2.98 (1.53–5.81)**
Non-Hispanic White	Ref	Ref	Ref	Ref
Other	0.64 (0.14–2.94)	0.45 (0.14–1.45)	0.93 (0.36–2.39)	0.7 (0.25–1.99)
Marital status				
Not Partnered	Ref	Ref	Ref	Ref
Partnered	2.31 (0.76–7.01)	3.04 (0.83–11.18)	1.00 (0.46–2.16)	1.29 (0.56–2.97)
Unknown	1.68 (0.09–32.17)	3.57 (0.23–54.61)	0.16 (0.06–0.37)***	0.21 (0.08–0.54)**
Insurance status				
Any private	Ref	Ref	Ref	Ref
Public insurance	3.86 (1.77–8.40)***	8.31 (3.41–20.25)***	2.43 (1.06–5.56)*	2.07 (0.95–4.53)
Uninsured	5.19 (0.53–51.21)	2.94 (0.33–26.51)	0.86 (0.22–3.32)	0.49 (0.12–2.09)
Yost Registry-Based Quintile				
Lowest	Ref	Ref	Ref	Ref
Low medium	0.35 (0.09–1.34)	0.56 (0.12–2.74)	1.09 (0.4–2.98)	0.84 (0.28–2.55)
Medium	0.29 (0.07–1.13)	0.33 (0.09–1.21)	0.61 (0.24–1.56)	0.71 (0.26–1.88)
Medium high	0.54 (0.17–1.67)	1.18 (0.35–4.05)	1.02 (0.43–2.43)	0.97 (0.41–2.30)
Highest	0.47 (0.07–3.2)	0.86 (0.16–4.62)	0.36 (0.13–1.01)	0.38 (0.14–1.03)
Clinical variables				
Stage at diagnosis^b^				
I		Ref		Ref
II		0.42 (0.12–1.52)		3.05 (1.23–7.56)*
III		11.89 (2.27–62.16)**		8.61 (3.27–22.66)***
Time from diagnosis to treatment				
≤ 8 weeks		Ref		Ref
> 8 weeks		1.08 (0.24–4.79)		2.58 (1.06–6.28)*

*TNBC* triple-negative breast cancer, *CI* confidence interval

^a^Model 1 adjusted for the sociodemographic variables. Model 2 adjusted for the clinical variables in addition to the sociodemographic variables. ^b^Derived from the American Joint Committee on Cancer (AJCC) staging system (seventh edition)

*p-value < 0.05; **p-value < 0.01; ***p-value < 0.001

Among women with TNBC, those diagnosed with Stage III cancers had higher hazards of dying (Model 2 HR: 11.89, CI 2.27–62.16) than women diagnosed with Stage I cancers. Additionally, having public insurance (Model 1 HR: 3.86, CI 1.77–8.40; Model 2 HR: 8.31, CI 3.41–20.25) was associated with higher hazard of dying compared to private insurance.

Among those with non-TNBC, non-Hispanic Black women had higher hazards of dying than non-Hispanic White women (Model 1 HR: 3.47, CI 1.77–6.80; Model 2 HR: 2.98, CI 1.53–5.81). Women with delays in treatment initiation (Model 2 HR: 2.58 CI 1.06–6.28) had worse survival than women without treatment delays. Women diagnosed with later-staged cancers had worse survival than women diagnosed with Stage I cancers (Model 2 Stage II—HR: 3.05, CI 1.23–7.56; Stage III—HR 8.61, CI 3.27–22.66). Having public insurance (Model 1 HR: 2.43, CI 1.06–5.56) was associated with significantly higher hazard of dying compared to private insurance in the model adjusted for sociodemographic variables but was no longer significant after further adjusting for clinical variables (Model 2).

### Landmark analysis

Eight patients survived 6 months or less; of these, seven were lost to follow-up, and one died from a cause other than breast cancer. Their diagnosis-to-treatment times ranged from 3.3 to 12.9 weeks. For patients with a diagnosis-to-treatment time exceeding 6 months (*n* = 11), survival times ranged from 31 to 67 months.

## Discussion

In this study, we examined the relationship between GCC, according to 2013 NCCN guidelines, and survival, by cancer subtype, among young adult women (under 40 years old) diagnosed with non-metastatic breast cancer in 2013. We found in univariate Cox models that non-GCC was associated with higher hazards of dying compared to GCC for TNBC and non-TNBC. However, in multivariable Cox models, the effect of sociodemographic and clinical factors on the association between receipt of NCCN GCC and survival differed by TNBC status. The results of the landmark analysis indicate that the findings are not affected by immortal time bias.

Among women with TNBC, we found in Kaplan–Meier survival curve, unadjusted Cox modeling, and multivariable Cox modeling adjusting for sociodemographic variables that those who did not receive GCC had significantly lower survival compared to those who received GCC. The finding is consistent with larger-scale studies of women with TNBC which indicated that GCC is likely associated with better survival among women of all ages [17, 18]. However, after the model was further adjusted for clinical variables, the association between receipt of GCC and survival was no longer statistically significant. The different outcomes may reflect the dominating strong association between stage at diagnosis and survival and/or the relatively small sample size for TNBC (*n* = 195). Stage at diagnosis was also associated with receipt of NCCN-defined GCC in a previous analysis of this POC dataset [12]. Our results for young adult women echo previous findings of late-stage diagnosis having much worse hazards for death among women of all ages with TNBC cancers [19]. In addition, we found an eightfold increase in hazards of death among women enrolled in public versus private insurance. This warrants additional investigation; future research should focus on the relationship between receipt of GCC and insurance status in women under 40 years old and aim to identify the determinants of the observed mortality difference.

Similar to analyses that examine the effect of GCC on survival among breast cancer patients in other age groups, which consistently find that adherence to GCC is associated with improved survival [18, 20]; in this study, among young adult women with non-TNBC, we found that GCC was associated with better survival. Although some studies have examined treatment received and AYA breast cancer survival, to our knowledge, this study is the first to examine the effect of GCC on survival among young adult breast cancer survivors.

In addition, our results among young adult women with non-TNBC are consistent with literature on breast cancer survival in all age groups: non-Hispanic Black women have worse survival from breast cancers [21], including AYA breast cancers [9]; later-stage cancers are associated with worse survival [19]. Although previous studies have found Medicaid insurance to be associated with worse survival than private insurance [22], among young adult women with non-TNBC, after adjusting for clinical variables, we found that public insurance was not associated with significantly worse survival than private insurance. Finally, our finding among women with non-TNBC that delays in treatment initiation are associated with worse survival, is consistent with research in AYAs [23]. Optimal timing of treatment initiation may be especially pertinent in women under 40 years old as AYA cancers are often more aggressive than cancers in other age groups, and delays in receipt of GCC may have unique effects on survival in this age group [1, 23].

### Limitations

Despite many strengths, this research also has some limitations. POC data lack details on rates of treatment completion and reasons for declining treatment. Such information could provide further insight into survival patterns in young adult women. Furthermore, the year of breast cancer diagnosis for these POC data was 2013, and there have been changes in the standard of care and NCCN guidelines for breast cancer treatment over the last decade. Our use of a strict definition of GCC which excluded those who received any therapy not specifically recommended by NCCN, may limit generalizability. Variations in NCCN-recommended treatment regimens may be appropriate for some women but POC does not collect data on factors that may be relevant to treatment selection for young adult women including acute toxicities, fertility and sexual dysfunction, and quality of life. This study is subject to the usual restrictions of secondary data research, including missing information and accuracy of data collection. We were unable to study women under 20 years old because there were none in the stratified random POC sample, so our findings are limited to breast cancer survivors 20–39 years old. Finally, this study does not investigate whether more intensive therapies result in better, similar, or worse survival than less intensive therapies. This may be important to consider, given that young adult women may undergo more intensive therapy for breast cancer [11], without survival benefits [24].

### Conclusion

In this study, we found that among women under 40 years old with TNBC or non-TNBC, GCC was associated with better survival. The results of this study support GCC in young adult women with breast cancers. Despite younger women receiving diagnoses of more aggressive subtypes of breast cancer, we found evidence that treatment according to guidelines (which are based on clinical factors) effectively promotes survival in this age group. Studies should continue to investigate the effect of treatment choice and guideline concordance on these factors. Additionally, research should focus on tracking young adult breast cancer survivors as they age, to consider the impact of different therapies on late toxicities, quality of life, and long-term survivorship.

## Data Availability

Instructions for requesting access to the Patterns of Care are available at https://healthcaredelivery.cancer.gov/poc/access.html.
